# Analysis of complex metabolic behavior through pathway decomposition

**DOI:** 10.1186/1752-0509-5-91

**Published:** 2011-06-03

**Authors:** Kuhn Ip, Caroline Colijn, Desmond S Lun

**Affiliations:** 1 Phenomics and Bioinformatics Research Centre, University of South Australia, Mawson Lakes, SA 5095, Australia; 2 School of Mathematics and Statistics, University of South Australia, Mawson Lakes, SA 5095, Australia; 3 Australian Centre for Plant Functional Genomics, University of South Australia, Mawson Lakes, SA 5095, Australia; 4 Center for Computational and Integrative Biology, Rutgers University, Camden, NJ 08102, USA; 5 Department of Computer Science, Rutgers University, Camden, NJ 08102, USA; 6 Department of Engineering Mathematics, University of Bristol, Bristol BS8 1TR, UK

## Abstract

**Background:**

Understanding complex systems through decomposition into simple interacting components is a pervasive paradigm throughout modern science and engineering. For cellular metabolism, complexity can be reduced by decomposition into pathways with particular biochemical functions, and the concept of elementary flux modes provides a systematic way for organizing metabolic networks into such pathways. While decomposition using elementary flux modes has proven to be a powerful tool for understanding and manipulating cellular metabolism, its utility, however, is severely limited since the number of modes in a network increases exponentially with its size.

**Results:**

Here, we present a new method for decomposition of metabolic flux distributions into elementary flux modes. Our method can easily operate on large, genome-scale networks since it does not require all relevant modes of the metabolic network to be generated. We illustrate the utility of our method for metabolic engineering of *Escherichia coli *and for understanding the survival of *Mycobacterium tuberculosis *(MTB) during infection.

**Conclusions:**

Our method can achieve computational time improvements exceeding 2000-fold and requires only several seconds to generate elementary mode decompositions on genome-scale networks. These improvements arise from not having to generate all relevant elementary modes prior to initiating the decomposition. The decompositions from our method are useful for understanding complex flux distributions and debugging genome-scale models.

## Background

Computational analysis of cellular metabolism has recently gained increasing prominence and importance. In particular, genome-scale computational models capturing stoichiometric and thermodynamic constraints have been published for over 30 organisms [1] ranging from relatively simple prokaryotes such as *E. coli*, to complex eukaryotes such as *Homo sapiens *[2,3]. The application of such computational models is dependent on their accuracy and the tools developed for their analysis. The maintenance of model accuracy, or debugging, is an ongoing process by which model predictions are validated against experimental observations and discrepancies are identified and corrected. This process is clearly linked with the use and analysis of the models. These computational models can be analyzed in a number of ways. Flux-balance analysis (FBA) and elementary mode analysis are perhaps two of the most popular and powerful.

FBA determines a distribution of steady-state reaction fluxes that satisfies the constraints of the model and that optimizes a biological objective function such as biomass or adenosine triphosphate (ATP) production [4]. With appropriate constraints and a biological objective, FBA has been shown to be an effective method for prediction of phenotypes associated with genetic manipulations such as knockouts [5,6] and of intracellular metabolic fluxes [7]. A significant application for FBA, therefore, is metabolic engineering-using computational predictions of metabolic phenotype under genetic manipulations to guide the engineering of metabolically optimized strains [8]-and computational methods have been devised to search the space of genetic manipulations *in silico *for those that yield the desired phenotype [9-13]. But, while FBA has had a number of successful applications, the method gives little insight into its predictions, hindering human understanding and model debugging.

Elementary mode analysis, on the other hand, yields no explicit predictions of metabolic behavior, and seeks primarily to allow understanding of an organism's metabolic capabilities. In elementary mode analysis, the elementary flux modes (EFMs) that define minimal sets of reactions capable of operating at steady state are generated [14]. Elementary flux modes formalize the concept of a biochemical pathway [15], and studying the modes associated with a given metabolic network has been shown to be effective for understanding its function, regulation, and robustness [16,17]. The principal drawback of elementary mode analysis is that the number of EFMs in a network suffers from a combinatorial explosion [18], and the use of complete sets of EFMs gives rise to problems with scalability when applied to genome-wide models [19]. For example, more than two million modes exist for a simple model of *E. coli *central metabolism consisting of 112 reactions [20], which increases to more than 26 million modes when the possible substrates are expanded [21]. *i*AF1260 [6], the most recent genome-scale metabolic network of *E. coli*, consists of 2077 reactions, and the number of reactions in *E. coli *metabolic models has increased steadily for the past two decades [22]. Thus, the computation time and memory storage required to enumerate all the EFMs of full and detailed genome-scale metabolic networks are prohibitively large.

For many applications, however, our understanding of particular metabolic functions of an organism, such as its ability to produce a desired metabolite, is of greater interest than our understanding of its full metabolic capabilities. In this case, it is not necessary to know all the EFMs of the network, but simply those that combine to give rise to a particular behavior. Previous approaches to this problem have relied on first computing all modes of the network [23,24]. More recently, an approach presented by de Figueiredo et al. [25], found biologically significant EFMs by identifying the K-shortest EFMs of the metabolic network without necessarily enumerating the whole set. Our motivation is similar in that we are not concerned with generating all the EFMs of a network. However, our approach differs greatly in that we wish to determine those elementary modes that combine to yield a given flux distribution. This flux distribution can consist of both measured and computationally predicted fluxes.

We present an algorithm to find the elementary modes that combine to produce any previously-specified flux distribution. This links the advantages of elementary mode analysis with the advantages of flux balance analysis, without requiring the prohibitive computation of all elementary modes. Our method is therefore applicable to genome-scale models and, because it can take as an input any flux distribution, it can be connected to particular experimental conditions [26] or genetic modifications [10].

To demonstrate the utility of our method and its applicability to genome-scale models, we apply it to genome-scale models of *E. coli *and MTB and show how its results can be used to interpret flux distributions related to the metabolic engineering of *E. coli *and to the survival of MTB during infection.

## Results and Discussion

### Overview

Our method takes a given steady-state metabolic flux distribution and the corresponding metabolic model, and produces a decomposition of the flux distribution into elementary flux modes. In this paper, we use flux distributions obtained by FBA, but our method can equally be applied to flux distributions obtained by alternative means, such as those derived from experimental measurement or obtained from genetic perturbation analysis methods such as MOMA [27] or ROOM [28]. As an elementary flux mode is itself a set of reactions operating at steady state, any flux distribution composed entirely of elementary flux modes must necessarily operate at steady state too. Therefore, an input flux distribution derived from experimental measurement may need to be balanced first to produce a steady state flux distribution, either by regression to fit the measured data or alternative means.

Our method operates by first selecting the reaction with non-zero flux of maximum magnitude from the given flux distribution. The algorithm then uses mixed-integer linear programming to find an elementary flux mode that both contains the selected reaction and is contained in the given distribution. The contribution of this elementary flux mode to the given distribution is determined before it is removed to give an updated flux distribution. The updated flux distribution is used as the input distribution for the next iteration of the algorithm, and this procedure is repeated until the updated flux distribution is zero (see Methods for details).

Elementary mode decompositions are not unique. Our goal is to assist in biological interpretation, and we are primarily interested in obtaining a valid decomposition rather than any specific decomposition. A valid decomposition will arise irrespective of the choice made at each iteration of the reaction with non-zero flux, but how this choice is made determines the specific decomposition. Our choice has some desirable properties. As the elementary flux mode found by the mixed-integer linear program includes the chosen reaction, the flux through this reaction will upper bound the contribution of the elementary flux mode. By selecting the reaction with non-zero flux of maximum magnitude, we avoid the possibility of generating many elementary flux modes with very small weightings and hence minimal contribution to the flux distribution. This choice also minimizes numerical instabilities arising from the calculations.

Although mixed-integer linear programming is NP-hard in general, some large mixed-integer linear programs (MILPs) can be solved with a modest amount of computation by solvers such as SCIP [29], IBM ILOG CPLEX (International Business Machines Corp., Armonk, New York), and Gurobi (Gurobi Optimization, Houston, Texas). In particular, our algorithm, implemented using Gurobi, successfully terminates in at most several seconds in all the genome-scale applications mentioned in this paper.

By contrast, previous approaches to decomposing flux distributions into elementary modes [23,24] have relied on first generating the complete set of relevant elementary modes, then calculating a weight distribution among these modes. Since elementary mode decompositions are not unique [23], the principal advantage of these previous approaches in comparison to ours is that they are capable of selecting a particular unique decomposition based on some criterion (typically the minimization of the Euclidean norm of the weight vector), while our approach simply generates a valid decomposition among all possibilities. It is not clear, however, that criteria for selecting the weight distribution, such as the minimization of the Euclidean norm, are biologically meaningful. As we will see with the examples in this paper, simply having a valid decomposition is in itself useful.

The principal drawback of these previous approaches is that generating the complete set of relevant elementary modes can be prohibitive, particularly for genome-scale models. To demonstrate this fact, we used efmtool [21] to efficiently generate the relevant elementary modes for the genome-scale model of MTB by Jamshidi and Palsson [30], *i*NJ661, for growth on Middlebrook 7H9-a standard growth medium for MTB. With the elementary modes generated by efmtool, we then applied the quadratic programming approach proposed by Schwartz and Kanehisa [23] to obtain a distribution of weights to assign to these modes. The total computational time of this approach was 34 minutes and 27 seconds.

By contrast, our approach generates a valid elementary mode decomposition consisting of 19 modes in 1.0 seconds-a computational time improvement exceeding 2000-fold. *i*NJ661 consists of 1,028 reactions, which is modest for current genome-scale models [1]. With the ever increasing size and complexity of genome-scale models [22] and the exponential manner in which the number of elementary modes increases with the size of the network, it follows that in some cases, the advantages offered by our approach may not simply be a several-thousand fold improvement in computational time, but rather the difference between practical feasibility and infeasibility.

Indeed, *i*NJ661v [31], a recent model that modifies *i*NJ661 with the aim of more accurately modeling MTB in *in vivo *infection, is only slightly larger than *i*NJ661, with 1,049 reactions. However, a more complex growth medium resulted in a significantly larger number of relevant elementary modes for the flux distribution obtained by FBA than that for *i*NJ661, and we were unable to generate them all using efmtool because of memory limitations. Using our decomposition method, we generated a valid elementary mode decomposition consisting of 27 modes in 2.5 seconds.

### *Metabolic engineering of *E. coli

To demonstrate the utility of our approach for metabolic engineering, we consider the metabolic engineering of *E. coli *for increased acetate production-a problem that has received attention owing to the value of acetic acid for its industrial and food uses [32]. Knockout strategies for increased production of acetate based on FBA have previously been reported by Lun et al. [10] using the genome-scale metabolic reconstruction of *E. coli *by Feist et al. [6], *i*AF1260. These knockout strategies were generated using GDLS (Genetic Design through Local Search) [10], an efficient heuristic for generating metabolic engineering strategies involving multiple knockouts from genome-scale models, extending the capabilities of the computationally-expensive optimal search proposed by OptKnock [9]. The strategies were chosen using FBA to have high predicted production of acetate whilst maintaining required energy production and growth to ensure viability. Specifically, a predicted non-growth-associated ATP maintenance (NGAM) flux of at least 8.39 h^-1 ^and a biomass flux of at least 0.05 h^-1 ^were required.

The proposed strategies make sense biologically [10] and include experimentally-tested knockouts for acetate production such as alcohol dehydrogenase and ATP synthase [32]. However, the cause for the increased acetate production is not immediately clear from the metabolic phenotype predicted by FBA alone. Table 1 shows the number of reactions that have been added to or removed from the flux distributions of the gene-knockout mutants when compared with the wild-type flux distribution. As we can see, the changes in the flux distributions are quite extensive and, in particular, they do not result simply from shunts around the blockages created by the gene deletions. The inherent structure of the network, and hence the mechanisms by which predicted metabolic behavior arises, is difficult to ascertain from the flux distribution as a whole. We, therefore, applied our decomposition algorithm to determine the elementary flux modes that make up the flux distributions under these knockout strategies. We took the best knockout strategies reported by Lun et al. [10] with numbers of knockouts ranging from 1 to 8. For the purposes of these strategies, a single knockout is considered to consist of all genes capable of catalyzing a reaction, i.e. the enzyme complex or all complexes with the same metabolic function.

**Table 1:** Number of changes to reactions used in each *E. coli *knockout mutant compared with the wild-type (WT)

Gene Knockouts	1	2	3	4	5	6	8
**Reactions with zero flux in WT and non-zero flux in mutant**	12	12	22	25	25	29	36

**Reactions with non-zero flux in WT and zero flux in mutant**	9	9	16	21	23	17	24

Table 2 shows the elementary flux modes we obtained. The flux distribution for each knockout mutant can be decomposed into two elementary modes, which together supply the energy and growth requirements of the organism. We emphasize that elementary mode decomposition will identify the structural components that are important to the metabolic phenotype. The focus on the NGAM and biomass components reflects their biological importance to the underlying biology of the model. The first mode only contributes to the required NGAM flux, and uses relatively few reactions. In comparison, the second mode is solely responsible for producing biomass and involves many more active reactions. This corresponds with the large number of metabolites required for biomass production. Furthermore, as noted by Stelling et al. [16], the unmodified *E. coli *metabolic network displays a degree of robustness as evidenced by the varied pathways by which biomass and NGAM are produced. The knockouts force flux onto alternative pathways for producing these necessary metabolic components, and these pathways produce acetate as a side-product. By finding and examining the elementary modes associated with the acetate-enhancing knockout strategies, we uncover the mechanism responsible for the increased acetate flux, which was not apparent from the FBA analysis.

**Table 2:** Elementary modes for acetate-producing *E. coli *knockout strategies^a^

Knockouts (Number in brackets, Separated by semi-colons)	Mode	Weight	Overall conversion (Acetate production in bold)	Biomass (g/mmol Glc)	NGAM (mmol/mmol Glc)	Number of reactions
**(0) **None	1	2.92	1.000 Glc + 0.005 K + 0.315 NH_4 _+ 0.028 Pi + 0.007 SO_4 _→ **0.785 Ac **+ 1.661 CO_2 _+ 0.766 EtOH + 1.073 H + 1.675 H_2 _+ 0.010 Succ	0.029	0.2	412
	
	2	7.08	1.000 Glc + 0.297 H_2_O + 0.004 K + 0.222 NH_4 _+ 0.020 Pi + 0.005 SO_4 _→ **0.849 Ac **+ 1.762 CO_2 _+ 0.835 EtOH + 1.051 H + 1.772 H_2 _+ 0.007 Succ	0.021	1.1	414
	
	Combined		10.000 Glc + 2.101 H_2_O + 0.041 K + 0.002 Mg_2 _+ 2.492 NH_4 _+ 0.222 Pi + 0.058 SO_4 _→** 8.301 Ac **+ 17.322 CO_2 _+ 8.152 EtOH + 10.578 H + 17.434 H_2 _+ 0.077 Succ	0.23	8.4	415

**(1) **frmA, adhE, adhP	1	4.18	1.000 Glc + 0.667 H_2_O → 0.667 12PPD-R + **1.333 Ac **+ 1.333 CO_2 _+ 1.333 H + 1.333 H_2_	0	2	38
	
	2	5.82	1.000 Glc + 0.004 K + 0.228 NH_4 _+ 0.020 Pi + 0.005 SO_4 _→ 0.554 12PPD-R + **1.121 Ac **+ 1.201 CO_2 _+ 1.330 H + 1.211 H_2 _+ 0.007 Succ	0.021	0.0045	414
	
	Combined		10.000 Glc + 2.788 H_2_O + 0.022 K + 1.327 NH_4 _+ 0.118 Pi + 0.031 SO_4 _→ 6.011 12PPD-R + **12.101 **Ac + 12.563 CO_2 _+ 13.313 H + 12.623 H_2 _+ 0.041 Succ	0.12	8.4	418

**(2) **mhpF, adhE; ydfG	1	4.07	1.000 Glc + 0.750 H_2_O → **1.500 Ac **+ 1.500 CO_2 _+ 0.750 EtOH + 1.500 H + 1.500 H_2_	0	2.1	54
	
	2	5.93	1.000 Glc + 0.047 H_2_O + 0.004 K + 0.237 NH_4 _+ 0.021 Pi + 0.005 SO_4 _→ **1.296 Ac **+ 1.306 CO_2 _+ 0.586 EtOH + 1.513 H + 1.353 H_2 _+ 0.007 Succ	0.022	0	417
	
	Combined		10.000 Glc + 3.331 H_2_O + 0.023 K + 0.001 Mg_2 _+ 1.406 NH_4 _+ 0.125 Pi + 0.033 SO_4 _→ **13.791 Ac **+ 13.848 CO_2 _+ 6.529 EtOH + 15.075 H + 14.128 H_2 _+ 0.044 Succ	0.13	8.4	418

**(3) **atpABCDEFGH; frmA, adhE, adhP; pgi	1	2.82	1.000 Glc + 0.444 H_2_O + 0.167 SO_4 _→ 0.444 12PPD-R + **1.556 Ac **+ 1.556 CO_2 _+ 1.222 H + 1.556 H_2 _+ 0.167 H_2_S	0	1.3	59
	
	2	7.18	1.000 Glc + 0.233 H_2_O + 0.001 K + 0.077 NH_4 _+ 0.007 Pi + 0.159 SO_4 _→ 0.408 12PPD-R + **1.495 Ac **+ 1.486 CO_2 _+ 1.251 H + 1.501 H_2 _+ 0.157 H_2_S + 0.002 Succ	0.0071	0.64	418
	
	Combined		10.000 Glc + 2.926 H_2_O + 0.009 K + 0.552 NH_4 _+ 0.049 Pi + 1.609 SO_4 _→ 4.181 12PPD-R + **15.118 Ac **+ 15.056 CO_2 _+ 12.431 H + 15.166 H_2 _+ 1.596 H_2_S + 0.017 Succ	0.051	8.4	421

**(4) **atpABCDEFGH; frmA, adhE, adhP; pgi; yahI, ybcF, yqeA	1	2.91	1.000 Glc + 0.444 H_2_O + 0.167 SO_4 _→ 0.444 12PPD-R + **1.556 Ac **+ 1.556 CO_2 _+ 1.222 H + 1.556 H_2 _+ 0.167 H_2_S	0	1.3	59
	
	2	7.09	1.000 Glc + 0.233 H2O + 0.001 K + 0.077 NH4 + 0.007 Pi + 0.159 SO4 → 0.408 12PPD-R + **1.495 Ac **+ 1.486 CO2 + 1.251 H + 1.501 H2 + 0.157 H2S + 0.002 Succ	0.0071	0.64	416
	
	Combined		10.000 Glc + 2.946 H_2_O + 0.009 K + 0.545 NH_4 _+ 0.049 Pi + 1.609 SO_4 _→ 4.185 12PPD-R + **15.124 Ac **+ 15.062 CO_2 _+ 12.428 H + 15.171 H_2 _+ 1.597 H_2_S + 0.017 Succ	0.051	8.4	419

**(5) **atpABCDEFGH; frmA, adhE, adhP; pgi; tnaA; yahI, ybcF, yqeA	1	2.87	1.000 Glc + 0.444 H2O + 0.167 SO4 → 0.444 12PPD-R + **1.556 Ac **+ 1.556 CO2 + 1.222 H + 1.556 H2 + 0.167 H2S	0	1.3	59
	
	2	7.13	1.000 Glc + 0.235 H2O + 0.001 K + 0.076 NH4 + 0.007 Pi + 0.158 SO4 → 0.408 12PPD-R + **1.496 Ac **+ 1.486 CO2 + 1.252 H + 1.501 H2 + 0.157 H2S + 0.002 Succ	0.0071	0.64	413
	
	Combined		10.000 Glc + 2.949 H_2_O + 0.009 K + 0.544 NH_4 _+ 0.049 Pi + 1.607 SO_4 _→ 4.185 12PPD-R + **15.128 Ac **+ 15.057 CO_2 _+ 12.435 H + 15.168 H_2 _+ 1.595 H_2_S + 0.017 Succ	0.05	8.4	417

**(6) **atpABCDEFGH; galP; mhpF, adhE; pgi; pitAB; ydfG	1	6.53	1.000 Glc + 0.762 H2O → 0.071 12PPD-R + **1.690 Ac **+ 1.357 CO2 + 0.524 EtOH + 1.690 H + 1.524 H2	0	1.3	77
	
	2	3.47	1.000 Glc + 0.280 H_2_O + 0.003 K + 0.156 NH_4 _+ 0.014 Pi + 0.004 SO_4 _→ 0.114 12PPD-R + **1.404 Ac **+ 1.304 CO_2 _+ 0.467 EtOH + 1.546 H + 1.388 H_2 _+ 0.005 Succ	0.014	0	424
	
	Combined		10.000 Glc + 5.945 H_2_O + 0.009 K + 0.542 NH_4 _+ 0.048 Pi + 0.013 SO_4 _→ 0.863 12PPD-R + **15.908 Ac **+ 13.386 CO_2 _+ 5.042 EtOH + 16.403 H + 14.766 H_2 _+ 0.017 Succ	0.05	8.4	427

**(8) **(sapD or trkA or trkG), (sapD or trkA or trkH), kch, kup; atpABCDEFGH; galP; guaB; mhpF, adhE; pgi; pitAB; ydfG	1	6.53	1.000 Glc + 0.762 H_2_O → 0.071 12PPD-R + **1.690 Ac **+ 1.357 CO_2 _+ 0.524 EtOH + 1.690 H + 1.524 H_2_	0	1.3	77
	
	2	3.47	1.000 Glc + 0.282 H_2_O + 0.003 K + 0.155 NH_4 _+ 0.014 Pi + 0.004 SO_4 _→ 0.114 12PPD-R + **1.406 Ac **+ 1.304 CO2 + 0.468 EtOH + 1.547 H + 1.389 H_2 _+ 0.005 Succ	0.014	0	424
	
	Combined		10.000 Glc + 5.953 H_2_O + 0.009 K + 0.539 NH_4 _+ 0.048 Pi + 0.013 SO_4 _→ 0.861 12PPD-R + **15.915 Ac **+ 13.387 CO_2 _+ 5.043 EtOH + 16.407 H + 14.769 H_2 _+ 0.017 Succ	0.05	8.4	427

^a^Metabolite abbreviations: 12PPD-R, (R)-Propane-1,2-diol; Ac, acetate; EtOH, ethanol; Glc, glucose; Pi, phosphate; Succ, succinate.

Most interestingly, when the modes are examined in terms of acetate production, we find that the most efficient modes are generally those that only contribute to NGAM flux. However, a biomass-producing mode is necessary to satisfy growth and viability requirements. Thus, the problem of selecting knockouts to maximize acetate production, given a limiting resource such as glucose, is not necessarily about finding a single optimal elementary mode. Rather, competing constraints demand that the chosen modes need to complement each other well. This can be seen in the reported decompositions for the various knockout mutants.

For example, of all the biomass-producing modes, the mode arising from the five-knockout mutant is the most efficient at 1.496 mmol of acetate per mmol of glucose. When more knockouts are allowed, the overall acetate production is improved despite a decrease in the acetate production efficiency of the biomass-producing mode. This decrease is offset by a shift towards using NGAM-producing modes with significantly more efficient production of acetate. For the six and eight knockout cases, the NGAM-producing mode generates 1.690 mmol of acetate per mmol of glucose.

Finally, as FBA does not necessarily yield a unique distribution, we implemented the recursive MILP algorithm from Lee et al. [33] to find alternate optima and then obtained decompositions for the corresponding flux distributions. Our results (not shown) indicate that the decomposition into two modes with distinct functions related to NGAM and biomass production is preserved for alternate optima. Thus, the decomposition of flux distributions into primarily NGAM-producing and biomass-producing modes is a robust quality.

### Understanding the survival of MTB during infection

To illustrate another application of our approach, we consider the utilization of the glyoxylate shunt in MTB. The glyoxylate shunt enzyme isocitrate lyase (ICL), present in MTB as two isoforms, is believed to be required by microorganisms to utilize fatty acids as a source of carbon and energy. This shunt has previously been shown to be required for the *in viv*o growth and virulence of MTB [34]. Since MTB is believed to subsist on fatty acids during infection [34,35], it is argued that, by removing ICL, MTB is no longer able to utilize fatty acids for carbon and energy and therefore unable to grow *in vivo*. Indeed, strains of MTB absent in both isoforms of ICL are unable to grow on fatty acid substrates and unable to survive in macrophages and mice [34]. Therefore, ICL has attracted significant attention as a promising drug target for treatment of MTB infection [36].

**Figure 1:**
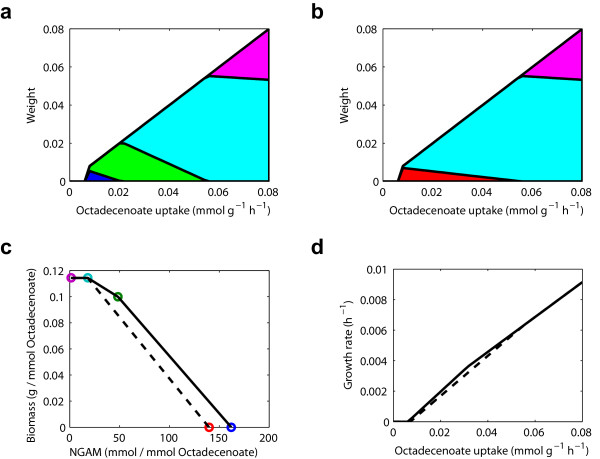
**Elementary modes used by MTB growing on octadecenoate as the sole carbon source with and without ICL**. Five unique elementary modes are identified overall by applying our decomposition method to three representative flux distributions, and these elementary modes are used to characterize optimal metabolic behavior for octadecenoate uptake varying from 0 to 0.08 mmol g^-1 ^h^-1^. The weights of these five elementary modes (colored blue, green, red, cyan, and magenta) as octadecenoate uptake varies are shown stacked **(a) **with and **(b) **without ICL present. The modes colored blue and green use ICL, while the remainder do not. Each mode is normalized so that the octadecenoate uptake of the mode is 1 mmol g^-1 ^h^-1^. **(c) **The biomass and NGAM generated by each mode with 1 mmol of octadecenoate. The region enclosed by the solid line is the space of achievable pairs of biomass and NGAM when ICL is present, while the region enclosed by the dotted line is that when ICL is not present. When octadecenoate is plentiful, the cyan and magenta modes can be used to meet the NGAM requirement and to produce biomass; when octadecenoate is more limited, the remaining modes are needed to meet the NGAM requirement and, with ICL present, this can be achieved more efficiently. **(d) **FBA-predicted growth rate under varying octadecenoate uptake with ICL present (solid line) and without it (dotted line).

It is possible, however, that given the robustness that is generally observed in metabolic networks [16], such a vital function would not simply rest on a single enzyme-even one present as two isoforms. Using our method, we can study the metabolic capabilities of MTB growing on fatty acid substrates. In particular, we determined the elementary modes used by MTB at differing uptake rates of octadecenoate using the genome-scale metabolic reconstruction of MTB by Beste et al. [37] (see Figure 1). We found that there exist modes that generate biomass and/or NGAM both using and not using ICL. At a given NGAM flux, the modes that use ICL generally produce biomass more efficiently than those that do not. However, for a given uptake of octadecanoate, the modes that produce the most biomass are those that do not use ICL. Therefore, the modes available to maximize the biomass production while maintaining a given NGAM requirement will depend on the supply flux of octadecenoate. When the supply is sufficiently high, the NGAM requirement is easily met by the high efficiency biomass producing modes that do not use ICL, but when it is lower, use of ICL allows the NGAM requirement to be met more efficiently and, hence, allows more biomass to be produced.

Under the assumption that the metabolic reconstruction by Beste et al. is correct, our analysis implies that MTB is capable of producing both carbon and energy from fatty acids without the use of ICL but, for lower uptake rates of fatty acids, ICL allows for more efficient utilization of the fatty acids. Indeed, we found that the optimal growth rate predicted by FBA differs only slightly with ICL present and without it (see Figure 1d). This presents the intriguing possibility that MTB possesses the metabolic capability to grow on fatty acids without ICL, but does not do so after the knockout of ICL because it has not yet undergone the adaptive evolution necessary to make use of this metabolic capability. This possibility is consistent with the work of Fong and Palsson [38] showing that the growth rate of gene deletion strains of *E. coli *can change significantly after undergoing adaptive evolution. The possible existence of such inactive routes for metabolizing fatty acids without the use of ICL in MTB has been discussed elsewhere [39] and, if true, would imply that MTB could rapidly evolve resistance to drugs inhibiting ICL.

Closer examination of the elementary modes reveals how MTB grows *in silico *without ICL and provides a testable means of confirming or rejecting the model's predictions. All the elementary modes that do not use ICL use malate synthase, the second of the two enzymes that form the glyoxylate shunt. In all of these modes, the full flux through malate synthase comes from HtrA, a gene predicted to code for 4-hydroxy-2-oxoglutarate aldolase to complete the hydroxyproline degradation pathway in MTB using the Bayesian method of Green and Karp [40]. HtrA supplies the glyoxylate that is used as a substrate by malate synthase. MTB and other mycobacteria have been observed to grow using hydroxyproline as a carbon source [41,42], suggesting that this pathway may indeed exist. Further studies confirming and characterizing this pathway will shed light on whether it does in fact provide MTB with a viable means of producing glyoxylate.

The elementary mode decomposition analysis we have presented sheds light on the mechanics of the FBA prediction that is difficult to obtain from the FBA results alone. Specifically, HtrA in combination with malate synthase can be used in GSMN-TB to generate biomass and NGAM. However, the space of possible biomass and NGAM production rates that can be achieved in this way is smaller than that which is possible using the glyoxylate shunt. At low octadecenoate uptake rates, this difference is important, leading to a lower biomass production to meet the NGAM requirement. Furthermore, it also demonstrates the utility of our method in identifying a potential source of the discrepancy between in silico predictions and observed experimental results. If growth is not experimentally possible, even after adaptive evolution, then it suggests that the model is incorrect and the likely error in the model comes from the inclusion of the predicted gene, HtrA.

## Conclusions

We have presented a method for decomposing a given flux distribution into a set of constituent elementary modes. In contrast to previous approaches, our method does not require the initial generation of a full set of elementary modes, which is often computationally demanding and, in some cases, computationally infeasible for practical purposes. In a moderately-sized instance, we observed a computational time improvement of over 2000-fold using our method.

Overall, we see that elementary mode analysis offers a great deal that flux-balance analysis alone does not. FBA yields predictions of overall metabolic behavior, while elementary mode analysis allows understanding of metabolic capabilities. By decomposing flux distributions obtained by FBA into elementary modes, we can gain insight into how metabolic networks achieve their predictions. We exploit modularity to decompose a complex entity into a simpler entity, which enables debugging and understanding and, ultimately, more sophisticated design and engineering.

## Methods

### Genome-scale FBA modeling

We work with the genome-scale model of *E. coli, i*AF1260. This model consists of three parts. First, from *m *metabolites and *n *reactions, we form an *m *× *n *stoichiometric matrix *S*, whose *ij*th element *S*_*ij *_is the stoichiometric coefficient of metabolite *i *in reaction *j*. Second, the flux distribution *v*, whose *j*th element *v*_*j *_is the flux though reaction *j*, is constrained by a lower bound vector *a*, whose *j*th element *a*_*j *_is the lower bound of the flux through reaction *j*, and an upper bound vector *b*, whose *j*th element *b*_*j *_> 0 is the upper bound of the flux through reaction *j*. Finally, the linear objective is formed by multiplying the fluxes by an objective vector *f*, whose *j*th element *f*_*j *_is the weight of reaction *j *in the biological objective. Thus, a biologically optimal flux distribution is obtained by solving(1)

### Elementary mode decomposition

For a given flux vector *ν*, we use *R*(*ν*) = {*i*:*ν*_*i *_≠ 0}to denote the set of indices of the reactions participating in *ν*. We define an elementary flux mode using the following two definitions [4].

#### Definition

A flux mode, or mode, in a metabolic network specified by a stoichiometric matrix *S *and lower and upper bound constraints *a *and *b *is a non-zero *n *× 1 vector *p *satisfying the following two conditions:

1. it is a valid steady-state flux distribution, i.e. *Sp *= 0;

2. irreversible reactions flow in the right direction, i.e. for all *j *such that *a*_*j *_≥ 0, we have *p*_*j *_≥ 0.

#### Definition

We say a flux mode is *elementary *if it is minimal among all flux modes, i.e. there does not exist any other flux mode such that *R*(*p*') ⊂ *R*(*p*).

Before stating the algorithm, we require one further definition.

#### Definition

We say a flux mode *p conforms *to a flux distribution *v *if *ν*_*j *_> 0 for all reactions *j *with *p*_*j *_> 0 and if *ν*_*j *_< 0 for all reactions *j *with *p*_*j *_< 0.

Our interest is in finding elementary modes that conform to a given flux distribution *v *since it ensures that *v *is decomposed into elementary modes that have reactions flowing in the same direction as *v*, for the purposes of biological interpretation.

Our algorithm takes as input a flux distribution *v *in the feasible set of optimization problem (1) and outputs an elementary mode decomposition for *v*, i.e. a set of elementary flux modes {*p*^(*k*)^} that conform to *v *and a corresponding set of positive weights {*w*_*k*_} such that . Suppose we have a flux distribution *v *that satisfies *Sv *= 0. Let *k*: = 1, and *v*^(*k*): ^= *v*. Choose some *j*_*k *_such that , and then solve the following mixed-integer linear program (MILP):(2)

where *M *is a large constant and sgn is the sign function, taking the value 1 if its argument is positive and -1 if its argument is negative. This MILP is similar to that used by de Figueiredo et al. [25] for finding the shortest elementary modes in a metabolic network. Our purpose, however, differs significantly: we seek to decompose a given flux distribution into constituent elementary modes. The specific choice of *j*_*k *_is unimportant: all choices such that  will generate a valid decomposition, though the specific decomposition achieved will likely vary with the choice. As discussed in the overview, we choose *j*_*k *_= argmax_*j*_|*v*_*j*_|.

Let (p*,q*) be an optimal solution. We then set *p*^(*k*)^: = p* and . Finally, we set *v*^(*k *+ 1)^: = *v*^(*k*) ^- *w*_*k*_*p*^(*k*)^. If *v*^(*k *+ 1) ^is the zero vector, then we are done. Otherwise, we choose *j*_*k + *1 _such that , increment *k *by one, and repeat the above procedure.

The following proposition establishes that this algorithm generates the desired output. In brief, the algorithm works because, at each iteration, the MILP finds a flux mode where *p*_*jk *_is non-zero and where the number of non-zero elements in the flux mode is minimized. Because the number of non-zero elements in the flux mode is minimized, the flux mode is minimal and, hence, elementary. Since each reaction with non-zero flux must participate in at least one elementary mode in the decomposition, it does not matter how *j*_*k *_is chosen, as long as . A valid decomposition will be generated regardless of how *j*_*k *_is chosen, though the particular decomposition that is generated among the non-unique possibilities will depend on this choice.

#### Proposition

The elementary mode decomposition algorithm stated above terminates after a finite number of iterations *K *and generates a set of elementary flux modes {*p*^(*k*)^} that conform to *v *and a corresponding set of positive weights {*w*_*k*_} such that .

#### Proof

First, to establish that each *p*^(*k*) ^is in fact a mode, we observe that any *p*^(*k*) ^generated as a solution of problem (2) will meet the steady state condition of the system. Problem (2) has a solution since  and *q *such that *q*_*j *_= 1 if *p*_*j *_≠ 0 and *q*_*j *_= 0 otherwise is in the feasible set of the problem. Further, by constraining the components of *p*^(*k*) ^to have the same sign as the corresponding elements of *v*, we ensure that irreversible reactions flow in the right direction since, for any *j*, if *a*_*j *_≥ 0 then *v*_*j *_≥ 0, which sets the constraint *p*_*j *_≥ 0 in problem (2).

Second, from the constraints of problem (2), we can see that *p*^(*k*) ^conforms to *v*.

Lastly, we establish that each *p*^(*k*) ^is minimal among all flux modes conforming to *v *and, therefore, elementary in the set of all such modes. We first observe that the optimal cost of problem (2) at iteration *k *is |*R *(*p*^(*k*)^)|. Suppose there exists a mode *p*' that conforms to *v *with *R*(*p'*) ⊂ *R*(*p*^(*k*)^). If , then we assume without loss of generality that , and (*p',q'*), where *q'*_*j *_= 1 if *p'*_*j *_≠ 0 and q'_j _= 0 otherwise, is in the feasible set of problem (2) and , in contradiction with *p*^(*k*) ^being an optimal solution. If , then let *p" *= *p*^(*k*) ^- *wp*', where . Now *p" *is a mode that conforms to *v *with *R*(*p'*) ⊂ *R*(*p*^(*k*)^) and (*p*", *q"*), where *q"j *= 1 if *p"j *≠ 0 and *q"j *= 0 otherwise, is in the feasible set of problem (2) and, resulting in the same contradiction. Hence there does not exist a mode *p' *that conforms to *v *with *R*(*p*') ⊂ *R*(*p*^(*k*)^), and we conclude that *p*^(*k*) ^is elementary.

It is straightforward to see that *w*_*k *_> 0 owing to its definition and that, after each iteration of the algorithm, |*R*(*v*^(*k*)^)| will decrease by at least 1, i.e. |*R*(*v*^(*k *+ 1)^)| < |*R*(*v*^(*k*)^)|. Thus the algorithm will terminate after a finite number of iterations *K *≤ |*R*(*v*)|.   □

### Characterization of optimal metabolic behavior using given elementary modes

When calculating the elementary mode decompositions for a range of related flux distributions, as in our MTB application, it is helpful to use only a subset of all elementary modes obtained, since it likely that a subset of the modes suffices to generate valid decompositions for all the distributions. To do so, we select a subset of *K *modes {*p*^(1)^, ..., *p*^(*k*)^} out of all those obtained and use the following approach to determine if they suffice to support all the flux distributions. We successively remove modes from the subset to arrive at one that is minimal in the sense that no additional modes can be removed and still support all the flux distributions.

We represent each elementary flux mode as a column vector in a matrix *P *=[*p*^(1) ^... *p*^(*K*)^] and define a non-negative weight vector *w = *[*w*_1_, ..., *w*_*K*_] such that a flux distribution *v *= *Pw*. Substitution of *v = Pw *into (1) gives a means of finding a biologically-optimal weight vector over the given set of elementary flux modes. Specifically, we solve(3)

If the biomass derived from solving (3) corresponds with that from (1), we conclude that the given elementary modes are sufficient to characterize the flux distribution of interest, and the given modes are utilized according to the weights w* obtained from the optimal solution of (3).

### Implementation of FBA and elementary mode decomposition

FBA and our elementary mode decomposition method were implemented using MATLAB R2010b and Gurobi 4.0. This implementation is available in additional file 1.

### Comparison to previous decomposition methods

We used Gurobi 4.0 to solve optimization problem (1) to find a biologically optimal flux distribution for *i*NJ661 growing on Middlebrook 7H9. The resulting distribution *v *contained 507 non-zero components. The reactions *j *for which *v*_*j *_= 0 were removed from the metabolic network, generating a reduced *S *that was used as input to efmtool. efmtool version 4.7.1 was used with default parameters in MATLAB R2010b to generate the elementary modes for the reduced metabolic network, resulting in a 507 × 131,558 matrix *P *containing all the elementary modes. Finally, the quadratic program

as proposed by Schwartz and Kanehisa [23] was solved using MOSEK 6.0.0.91 (MOSEK ApS, Copenhagen, Denmark).

efmtool generated the 131,558 elementary modes for the network in 1 minutes 48 seconds, while the quadratic optimization step took 32 minutes and 39 seconds, resulting in a total computational time of 34 minutes and 27 seconds. Computations were carried out on the Mac OS X 10.6.4 platform using a computer with an Intel Core 2 Duo 2.53 GHz processor with 4 GB of RAM.

For *i*NJ661v, the biologically optimal flux distribution *v *obtained by solving optimization problem (1) contained 505 non-zero components. Again, a reduced *S *was generated by removing the reactions *j *for which *v*_*j *_= 0, and the result used as input to efmtool. With a maximum heap size of 4 GB, efmtool failed before generating all elementary modes, with 657,447 modes generated.

## Authors' contributions

DSL and CC conceived the project. DSL and KI designed and implemented the method and performed the computational experiments. DSL, CC, and KI wrote the paper. All authors have read and approved the final manuscript.

## Supplementary Material

Additional file 1 Source code for elementary mode decomposition method implemented using MATLAB and Gurobi Click here for file
